# Natural Biased Coin Encoded in the Genome Determines Cell Strategy

**DOI:** 10.1371/journal.pone.0103569

**Published:** 2014-08-04

**Authors:** Faezeh Dorri, Hamid Mahini, Ali Sharifi-Zarchi, Mehdi Totonchi, Ruzbeh Tusserkani, Hamid Pezeshk, Mehdi Sadeghi

**Affiliations:** 1 Institute of Biochemistry and Biophysics, University of Tehran, Tehran, Iran; 2 Department of Computer Science, University of Maryland, College Park, Maryland, United States of America; 3 Department of Stem Cells and Developmental Biology at Cell Science Research Center, Royan Institute for Stem Cell Biology and Technology, ACECR, Tehran, Iran; 4 Department of Genetics at Reproductive Biomedicine Research Center, Royan Institute for Reproductive Biomedicine, ACECR, Tehran, Iran; 5 School of Computer Science, Institute for Research in Fundamental Sciences, Tehran, Iran; 6 School of Mathematics, Statistics and Computer Science, College of Science, University of Tehran, Tehran, Iran; 7 School of Biological Science, Institute for Research in Fundamental Sciences, Tehran, Iran; 8 National Institute of Genetic Engineering and Biotechnology, Tehran, Iran; Arizona State University, United States of America

## Abstract

Decision making at a cellular level determines different fates for isogenic cells. However, it is not yet clear how rational decisions are encoded in the genome, how they are transmitted to their offspring, and whether they evolve and become optimized throughout generations. In this paper, we use a game theoretic approach to explain how rational decisions are made in the presence of cooperators and competitors. Our results suggest the existence of an internal switch that operates as a biased coin. The biased coin is, in fact, a biochemical bistable network of interacting genes that can flip to one of its stable states in response to different environmental stimuli. We present a framework to describe how the positions of attractors in such a gene regulatory network correspond to the behavior of a rational player in a competing environment. We evaluate our model by considering lysis/lysogeny decision making of bacteriophage lambda in *E. coli*.

## Introduction

How do organisms adapt in order to survive in ever-changing environments? In this process, one important factor is mutation that affects the survival of organisms through proper genotypic changes. In a classical view, natural selection chooses the best-fit phenotype in the environment, and the next generation inherits the genotype associated with it. In a dynamic environment, genotype correction can be thought of an organism's strategy to survive. The fact that evolution occurs in an environment containing competing organisms, each playing its own optimized strategies, further complicates the picture.

In terms of time, biological evolution is a process that stabilizes after several trial and errors. However, some environmental fluctuations happen so quickly that there is not enough time for the organism to adapt itself through appropriate mutations. Organisms may face challenges caused by fluctuations in extracellular conditions. Early studies focused on the relationship between these environmental fluctuations and genetic diversities [1]–[4]. Other studies have shown that phenotypic variation exists, not as a consequence of underlying heritable genetic variation, but as an independent way of adapting to an ever-changing environment [5]–[9]. They discovered that cells with the same genotype can play different strategies and exhibit different phenotypes even when they are living in an identical environment.

From an extracellular point of view, phenotype variability as a risk-reducing strategy has been modeled by evolutionary game theory [10]–[16]. In this model, individuals with the same genotype compete for a longer life and more descendants by using various strategies. These strategies are interpreted as being different phenotypes. The fitness of an individual depends on the benefits and costs accrued by that individual in the presence of others. The pattern of phenotypic variations, which cannot be invaded by any alternative phenotypes, is described and predicted using the evolutionarily stable strategy (ESS) [10]. In ESS, the ratio of different phenotypes is considered as a mixed strategy which does not include changes at the level of the genotype. This concept has been successfully applied to explain why bacterial RNA-phage has different frequencies of Phi6 and PhiH2 phenotypes [11]. It has been shown that the fitness of phenotypes in RNA-phage generates a payoff matrix which is similar to the payoff matrix of the prisoner's dilemma problem. Furthermore, evolutionary game theory has provides an appropriate framework to learn important evolutionary phenomena such as altruistic behavior [12], [13], the evolution of sex ratio [14], pathogen-host interaction [15], and the rate and quantity of ATP production in different pathways of ATP synthesis in yeast [16].

From an intracellular point of view, there exist several biochemical reactions underlying phenotypic variation. The study of these intracellular functions is associated with quantitative genetic analysis such as gene regulatory networks [17], [18]. Gene regulatory networks play an important role in controlling the cellular behavior in varying environments. The structure and features of gene regulatory networks are evolved as a result of adaptation to fluctuating environment [19].

A regulatory network of genes with positive and negative feedback loops creates a potential landscape with different attractor states and bifurcation points [20]. Moreover, it explains reshaping of the landscape based on alterations in network parameters. In the 1940s, Waddington provided a basis to explain how the cells of an organism evolve differently during embryonic development [21]. He introduced the term *epigenetic landscape* and portrayed it as a marble rolling down a mountain with different valleys that eventually comes to rest at the lowest point which represents the ultimate fate of the cells. The valleys of the landscape represent stable attractor states while the other less stable states represent transient states of the early embryonic or progenitor cells [20]. The location and the shape of the attractor states define the natural probabilities of different biological decisions. It is not entirely clear how a qualitative picture of a landscape can be quantified and how the structure of the landscape is encoded in the genome.

**Figure 1:**
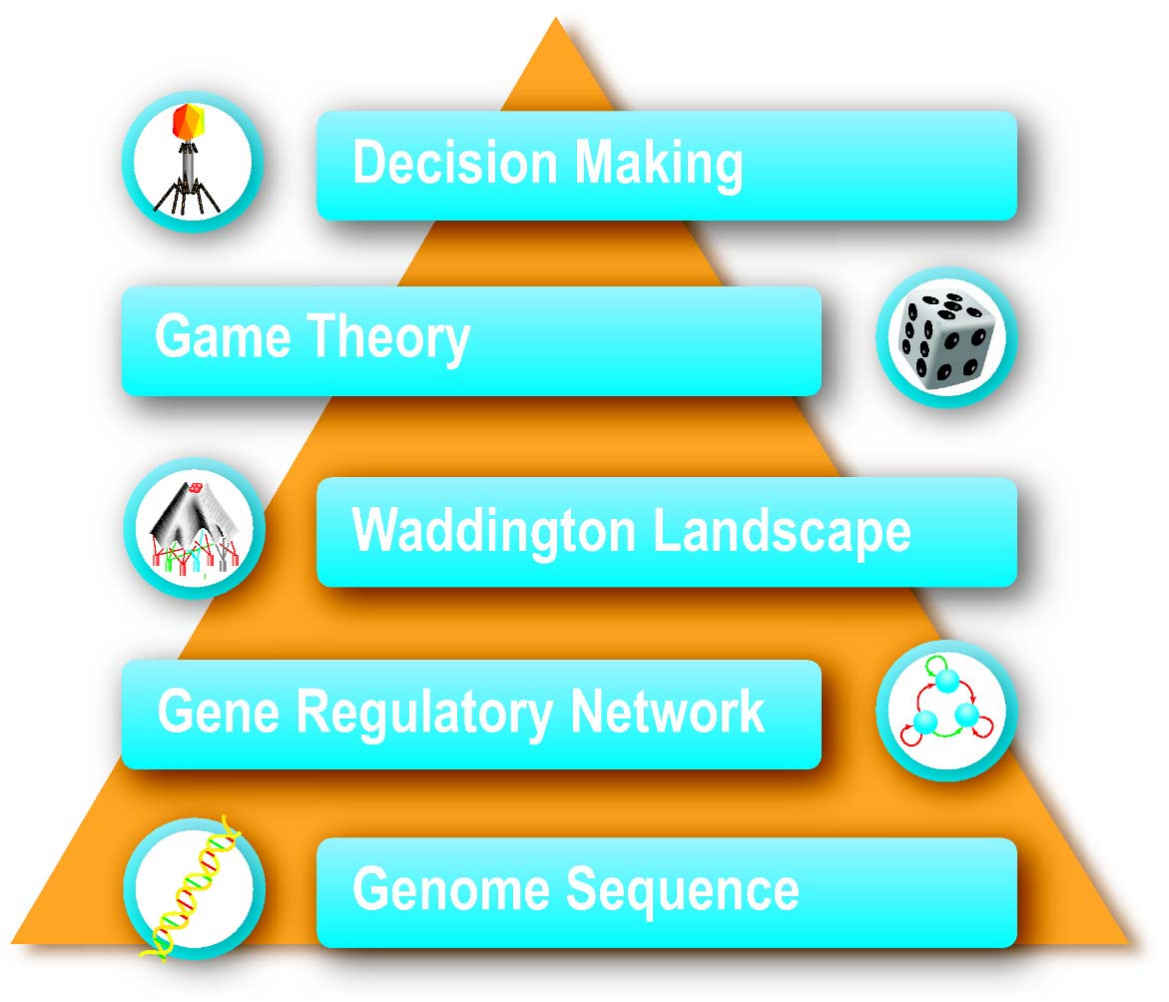
The proposed framework. The framework that links the game theoretic perspective of decision making in a living organism and Waddington's perspective of the underlying gene sequence.

We present a framework that combines the cooperative and competitive decision making of a living organism with its underlying intracellular gene regulatory network (see Figure 1). In this framework, game theoretic methods are applied to model the strategies of various living organisms. We show that the natural probabilities of organisms' decisions are fine-tuned to increase their chance of survival. Then, we argue that the location and the shape of the attractors in the Waddington landscape define the natural probabilities of different biological organisms, while the location and shape of attractors are characterized by the structure of the gene regulatory network. Altogether, we propose a framework that describes quantitatively how a gene regulatory network directs a cell to behave in a manner that is similar to that of a rational player in a game. This implies that the probability distribution of a rational decision, if we model a living organism as a cooperative and competitive decision maker, matches the probability distribution over stable states of its underlying gene regulatory network.

Our framework is supported by experimental data from one of the well-studied biological cases of phenotypic variation, the infection of *E. coli* with bacteriophage lambda. After *E. coli* is infected by bacteriophage lambda, the virus chooses between lysogenic or lytic pathways [22]–[24]. In the lysogenic mode, the virus's genome is inserted into the bacterial genome and is replicated along with the bacterial genome; viral particles are not produced. In the lytic pathway, the viral genome is replicated independently of the bacterial genome, viral particles are produced within the bacterial cell, the membrane of the host cell is lysed and the particles are released into the environment. Hence they can resume the infection cycle in other bacterial cells.

The decision between lysogenic and lytic pathways contains a trade-off between safe maintenance of the viral genome within the bacterial host genome and increased bacteriophages production. Similar counter-intuitive situations exist where individuals sacrifice themselves and cooperate to win the overall game [25]. At the molecular level, the bacteriophage lambda gene regulatory network acts like a switch with two attractor states. This bistable network is the same as a biased coin that is falling on either side with different probabilities according to the environmental conditions. We demonstrate how the gene regulatory network directs phages to behave in a manner very comparable to what we expect from a rational player in a game.

## Description of the Model

Representing living organisms as cooperative and competitive agents, game theory provides an elegant mathematical model to describe the behavior of a rational player. Consider an individual that makes a decision by selecting an action from a set of all possible actions 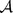. The strategy of the individual can be seen as a probability distribution over all possible actions of the finite set 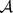. In other words, the strategy of the individual can be defined by a vector 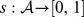, where 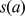 shows the probability of action 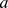 and 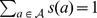. The strategy is pure if 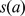 has only values 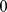 or 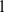. It is mixed if 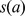 accepts all values in the interval [0, 1]. The *utility* of an individual represents how efficient its behavior is with respect to the environment. In general, each individual's utility depends on the state of the environment and strategies of all other individuals in the game. Therefore, the utility of individual 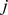 is usually represented as a function 
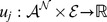
 where 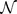 is the set of size 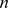 of all individuals and 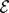 is the set of all possible environmental states. In this paper, we consider a situation where the effects of other strategies are implicit, and assume the utility of an individual only depends on the environmental state, i.e., 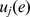 represents the utility of individual 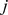 when the state of the environment is 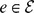.

As a consequence, the utility of an action 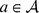 depends on the state of the environment. However, an individual only detects internal signals through its biochemical network and thus predicts the environmental state. Let 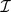 be the set of all possible internal states and 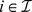 be an instance of it. Assume 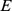 and 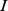 are two random variables defining the environmental state and internal state respectively. An individual detects internal state 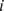 and infers the environmental state by determining posterior probabilities 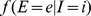, for each 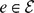. To summarize the system dynamics, we express it in the following iterative steps. First, the environmental state 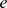 affects each individual's internal state. Second, each individual infers the environmental state based on its internal signals. Third, each individual estimates the expected utility of each action 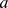 based on its internal state. Fourth, each individual chooses its mixed strategy based on the expected utility of each action. At last, all individuals' strategies determine the future environmental state. It is worth mentioning that while individuals' strategies affect the environmental state, we do not aim to model these effects in this paper.

From an intracellular point of view, the gene regulatory network is responsible for determining an organism's behavior. We represent the gene regulatory network 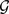 as a set of 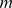 vertices 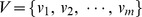 and a set of weights 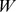 where 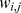 is the effect of gene 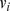 on gene 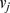. A positive value for 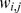 means 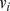 initiates the expression of 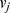 and a negative value for 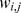 means 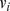 inhibits the expression of 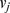. Every gene regulatory network characterizes a dynamical system which can be represented by a set of differential equations [26], [27]. Let 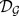 be the set of all attractors of the dynamical system of gene regulatory network 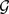. A dynamical system in good conditions ends up in one of its attractor states as time goes to infinity and each attractor state induces a specific action. In this model, the action of a cell deterministically depends on its initial conditions. However, finding the exact initial conditions of a cell in a noisy environment is impractical. Therefore, we assign different probabilities to different attractor states by considering stochastic initial states. In particular, a probability distribution 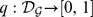 is assigned to a dynamical system to describe the state of the system as times goes by. For every 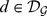 the value 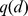 shows the probability that a system with a random starting point ends up in attractor state 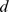.

## Results

Let us consider an organism which receives internal signals and selects between all its possible actions. The probability distribution 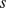 will be the strategy of a rational player in this situation. We demonstrate that for every internal, signal the weights of edges in the gene regulatory network are changed such that the probability distribution 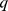 of the corresponding dynamical system is almost the same as the mixed strategy 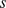 of the corresponding game.

### Gene regulatory network of the lysis/lysogeny decision

After the *E. coli* has been infected with bacteriophage lambda, a decision is made between either the death of the host (lysis) or viral dormancy (lysogeny) [28], [29]. Multiple internal and environmental signals arising during the decision making process affect the biochemical network and consequently, the fate of phages. These signals include the number of infecting phages, the metabolic state of the bacterium, the position of the infecting phages on the bacterium surface, and the bacterium size [22]–[24], [30]–[32]. To model the decision making process of bacteriophage lambda, some parameters are more prominent in the final state of bacteriophage. The number of phages infecting a bacterium (multiplicity of infection; 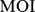) has long been known to affect the final decision of the bacteriophage [22], [30]–[32]. In addition, recent results represent the volume of the infected bacterium as an important parameter in the decision-making process [23], [24]. Recent results also show the relation between the concentration of phages in the host bacterium and the phage's final fate [23], [24].

**Figure 2:**
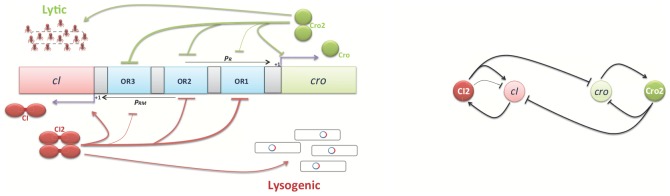
Regulatory network of bacteriophage lambda. Left: Genes and operators which are involved in lysogenic and lytic process of bacteriophage Lambda. Right: Simplified regulatory network of 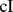 and 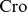 in bacteriophage lambda.

From intracellular perspective, the decision making of bacteriophage lambda is linked to the competitive gene expression of 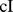 and 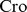 (see Figure 2). The regulatory circuit chooses between two outcomes including 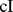 and 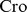 genes. The expression of the 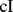 gene will lead to a lysogeny decision, and 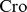 expression is followed by lysis [30]–[34]. These two genes are about 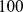 nucleotides far apart in the genome, and the area between them consists of the promoter of 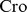 (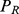), the promoter of 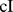 (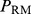), and three operator sites 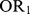, 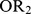, and 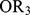. The operator sites 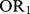 and 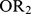 are located in 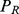, and operator site 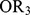 is located in 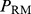 [35] (see Figure 2). Dimer 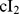 has a strong binding affinity to 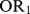. Dimer 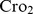 acts contrarily with the highest binding affinity to 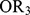. Binding of 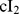 to 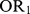 and 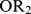 inhibits 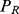 (down regulating 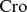) and has a positive effect on 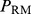 (auto-activating), while binding of 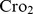 to the operator sites has a negative effect on the expression of the two genes.

### Rational decision making perspective

After the infection of *E. coli* by bacteriophage lambda, a decision is made between lysis and lysogeny. A rational decision infers the environmental state (the average 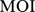), based on internal signals and increases the probability of lysogeny in a higher than expected average 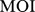. The host bacterium 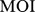, size, and concentration as internal signals define the phage fate. We calculate the probability distribution of estimated average 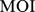 in different situations and describe the effect of host bacterium 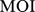, size and the concentration on it. Since the probability of each action, 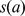, highly depends on estimated average 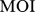, a higher estimated average 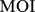 results in a higher probability of lysogeny.

**Figure 3:**
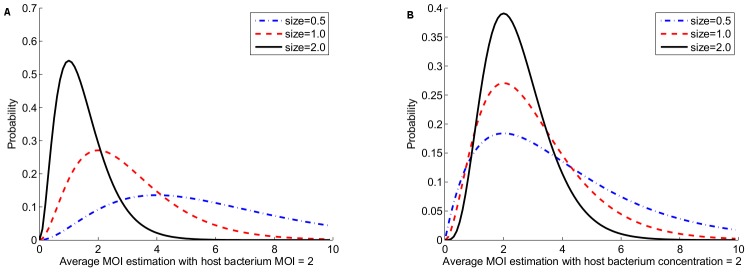
The probability distribution of estimated average 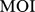. (A) The host bacteria have the same 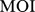, but different sizes. (B) The host bacteria have the same phage concentration, but different sizes.

Let 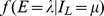 be the probability that the average 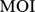 equals 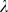, given host bacterium size 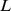 and host bacterium 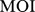 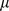. In other words, we are infering the enviromental state (the avarege 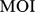), with respect to the internal signals (host bacteriom size and 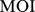). The probability 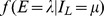 is calculated in Figure 3 for a fixed 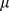 and different host bacterium sizes. The estimated average 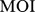 is highly correlated with the number of phages in a fixed size host bacterium.

The probability 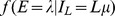 is shown in Figure 3 for a specific host bacterium concentration and different host bacterium sizes. Figure 3 demonstrates the effect of the host bacterium size on the estimation of 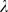 for the same host bacterium concentration. The expected value of estimated average 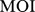 remains unchanged for the same host bacterium concentrations. However, it has been shown that for the same concentration, the variance decreases as the size of the host bacterium increases. This implies that increasing the size of the host bacterium decreases the probability of having a high environmental 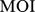. In conclusion, we expect different reactions from a rational player in situations with the same host bacterium concentration but different host bacterium sizes.

**Figure 4:**
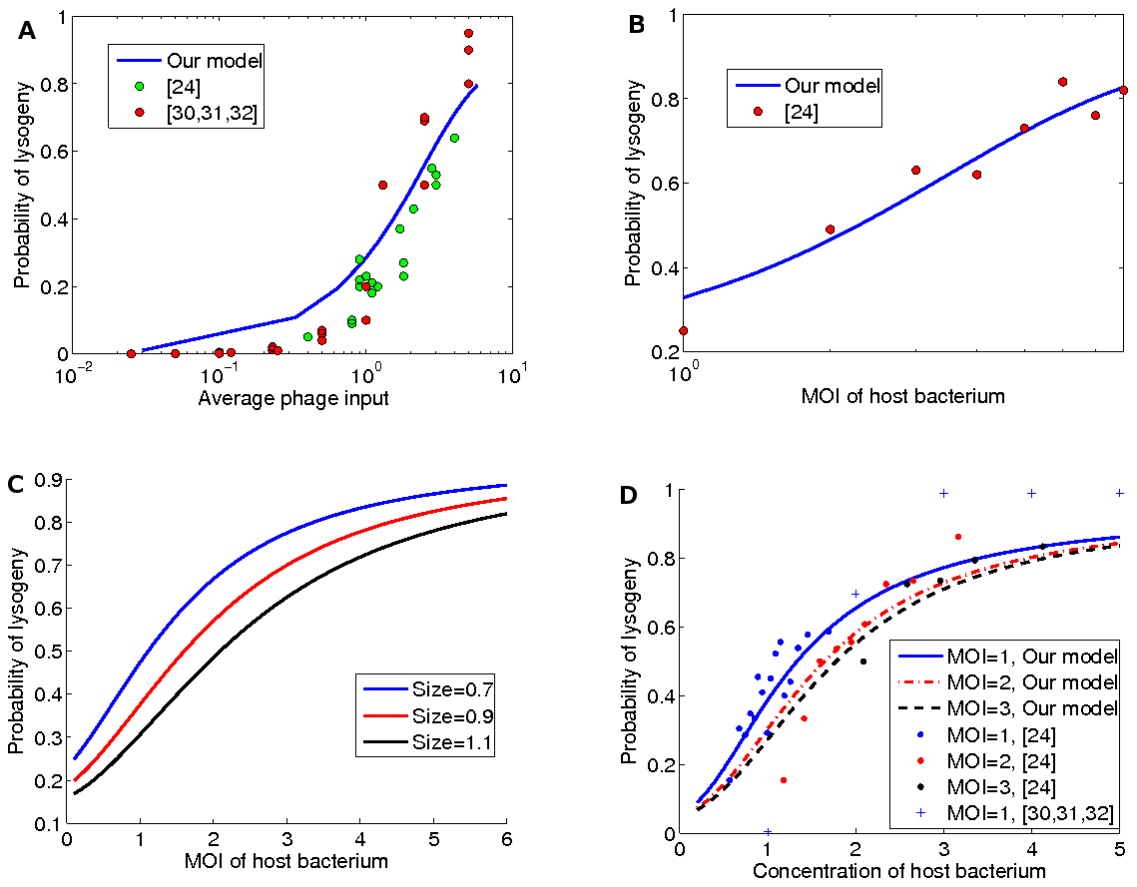
The probability of lysogeny as a function of the avarege phage input, the host bacterium 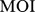, and the host bacterium concentration. (A) The average phage input is the total number of phages divided by the total number of bacteria and it is shown in logarithmic scale. Red circles show phages behavior based on the experimental data of [30]–[32]. Green circles show phages behavior based on the experimental data of [24]. Blue curve shows our estimation of rational players reactions with a RMSE of 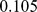. (B) The probability of entering lysogeny as a function of the host bacterium 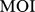. The host bacterium 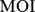 is shown in logarithmic scale. Red circles show phages behavior base on the experiments of [24]. Blue curve shows the estimation of rational players reactions with an RMSE of 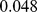. (C) The effect of the size of host bacterium on the probability of lysogeny. The results are displayed for the host bacterium sizes 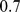, 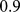, and 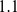. (D) The probability of lysogeny versus host bacterium concentration with different host bacterium 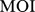. Blue, red, and black lines represent the estimations based on our model for 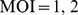, and 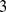 respectively. Blue, red, and black points show the experimental results of [24] for 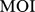 = 1, 2, and 3 respectively. Blue plus shows the experimental result of [30]–[32] for 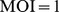. Note that our estimations have an RMSE of 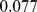.

In Figure 4, we use the experimental data of [30]–[32] and [24] to analyze the phages' behavior. The experimental data of [30]–[32] measures the rate of lysogeny versus the average 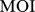. The same data has been used in [36]. The second experimental data set comes from [24]. This work measures both the probability of lysogeny based on the host bacterium 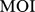 and also the rate of lysogeny based on the average 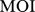 [24]. We calculate the expected probability 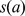 for both the lysis and the lysogeny actions. For a given average 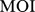, we integrate over all possible host bacterium sizes and different 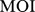
s to calculate the expected probability of lysogeny. The probability distribution of the host bacterium 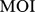 is modeled by a Poisson distribution for a given average 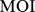. Figure 4 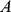 shows the expected probability of entering lysogeny as a function of the average 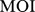. This figure clearly illustrates that the behavior of phages is remarkably close to the behavior of rational players. We use the root-mean-square error (RMSE) to measure the accuracy of the proposed model and obtain an RMSE of 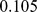.

We use the experimental data of [24] to verify our results. The data in [24] estimates the probability of entering lysogeny based on the host bacterium 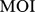. We compute the expected probability 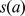 for both actions lysis and lysogeny. For a given host bacterium 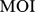, we integrate over all sizes of the host bacterium to calculate the expected probability of lysogeny (see Figure 4). An RMSE of 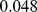 confirms that the behavior of a rational player is almost the same as the bacteriophages' behavior.

Figure 4 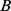 also shows the probability of lysogeny for different host bacterium 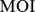
s. Phages tend to lysogenize as the 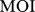 increases, since the estimation of average 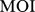 is an increasing function of the host bacterium 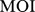. Moreover, Figure 4 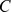 indicates that the probability of lysogeny decreases as the size of the host bacterium increases. In fact, the growth of the host bacterium size reduces both the concentration of phages and the estimated average 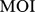.

The probability of lysogeny in the different concentration of phages inside the host bacterium is illustrated in Figure 4 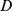. As we have discussed, the size of the host bacterium is one of the most important variables in the lysis/lysogeny decision [23]. Nevertheless, in the same concentration of the host bacterium, the phages in the smaller host bacterium show more tendency to lysogenize [24]. Our model's assessment of phage behavior with an RMSE of 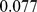 matches the results of [24] as shown in Figure 4. Let us recall that the variance of average 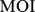 estimation is higher for a small bacterium than a large bacterium (see Figure 3). Hence, in a fixed concentration, it is more likely to have a higher estimated avarage 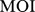 in a smaller host bacterium. This means in the same concentration, the probability of lysogeny increases as the size of the host bacterium decreases.

### Intracellular perspective

**Figure 5:**
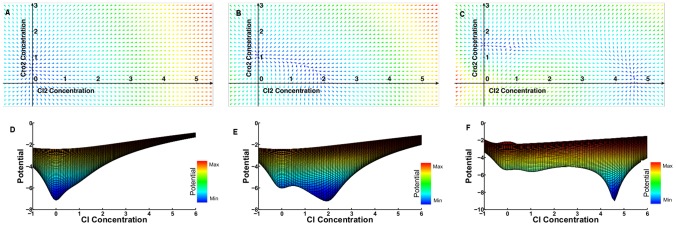
The effect of 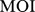 on the Waddington landscape of phages in the lysis/lysogeny decision making. Three different values of 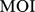 are presented in three rows. (A, B, C) The 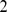-D force-field representation for 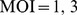 and 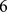 respectively. The concentration of 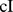 and 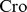 free-monomers are assigned to the 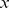-axis and 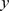-axis respectively. The small arrows are directed toward the attractor states which are shown by the blue areas. (D, E, F) The 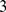-D potential function representation for 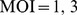 and 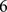 respectively. The 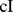 concentration, the value of the potential function, and the 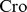 concentration are shown by 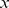-axis, 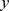-axis, and 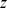-axis respectively. The attractor states are displayed as the local minimum of the potential function.

Analytical and numerical methods for solving differential equations will enable us to derive a set of steady state equations for a given system. To analyze this dynamic system, however, we would need more advanced methods. For this reason, we approach the problem from a Waddington perspective. As shown in Figure 5, the Waddington landscape of the phages' decision-making varies with the number of phages infecting the same bacterium. In the case of a single phage infection, there is one attractor state with high 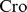 concentration that results in a lysis decision. Thus, the majority of the bacteria undergo lysis after a single infection (although there is a small probability for lysogeny due to the internal noise). A new attractor state with high 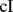 concentration is distinguishable at 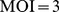. This results in a lysogeny decision. At 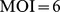, although there is still a vestige of the lysis attractor, but the deeper valley of the other attractor shows a higher likelihood of attracting the phages toward lysogeny. Video S1 describing changes of attractor points in the Waddinton landscape, for the host bacterium 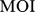 from 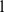 to 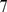, is provided in the supplementary material.

**Figure 6:**
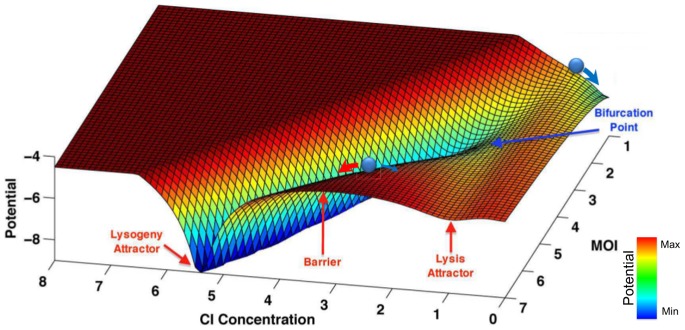
The Waddington landscape of lysis/lysogeny in different 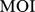. The Waddington landscape changes from 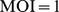 to 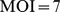. The back-to-front axis represents increasing 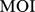 values, the right-to-left axis shows different 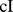 protein concentrations, and the vertical axis shows the pseudo-potential function. At 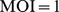 there is almost one valley with 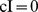 which represents a lysis attractor state. Increasing 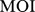 results in a bifurcation and at 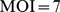 there are two distinct valleys with low (lysis) and high (lysogeny) 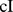 concentrations. The potential values higher than 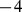 are truncated for better visualization.

To visualize the proposed landscape similar to the well-known drawing of Waddington envisioned in his book [21], the results of different 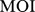 values are integrated in a 3D contour (Figure 6). According to the Waddington's original drawing, the vertical axis of the diagram represents the potential function and the back-to-front axis represents time [20]. For the lysis/lysogeny decision, we assign different values of 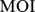 to the back-to-front axis. The left-to-right axis of Waddington's original diagram is the output of the system. It can be either the gene expression levels or phenotypic markers, whichever suitably distinguish between different cell types. The concentration level of the 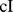 gene is chosen for this purpose in Figure 6. The attractors with low 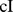 concentration represent the lysis state, and the attractor with high 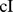 concentration represents the lysogeny state.

The Waddington landscape of a host bacterium with different 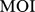 is shown in Figure 6. In a single phage infection, (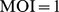), the bacterium goes for lysis in the backside of the landscape. There is only one lysis valley with 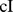 concentration close to 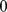. Due to the stochasticity of the system, there is a low probability for bacterium to undergo lysogeny after a single infection. As 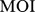 increases, the single valley bifurcates and a new attractor for lysogeny appears in high 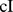 concentrations. The front-side of the landscape shows what happens at 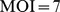. At this point, the channel for lysogeny is deeper (more stable) and wider (more probable) than the lysis. The high-potential barrier separating the two valleys represents the unstable states which the cells rarely pass. It justifies why the bacterium cannot easily change the outcome from lysis to lysogeny or vice versa.

**Figure 7:**
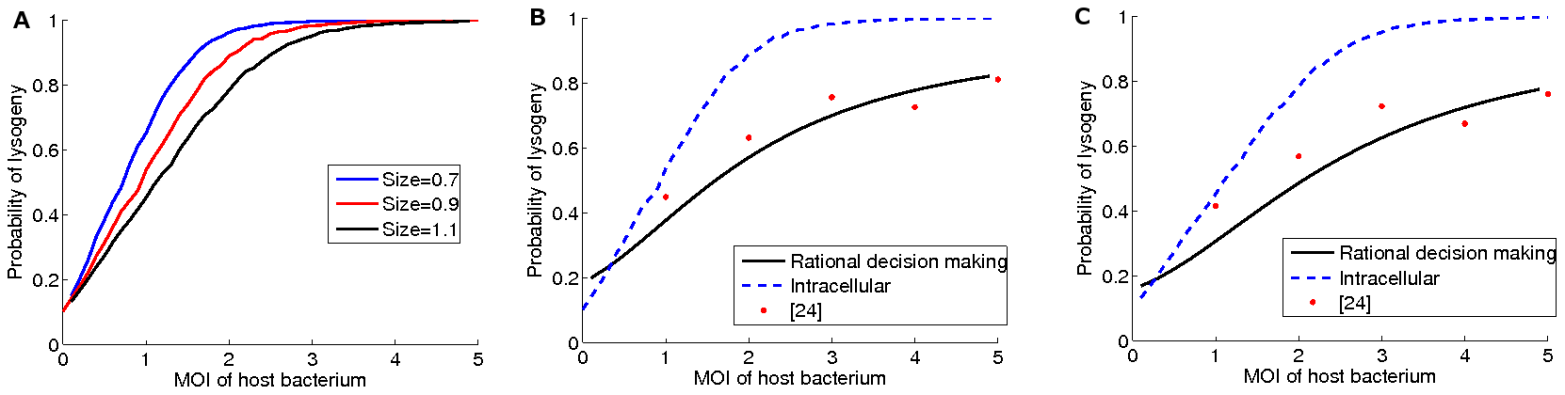
The probability of lysogeny in different models. (A) Lysogeny decision-making probability based on Waddington's model affected by bacterium size. The horizontal axis is 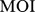 value and the vertical axis shows the probability of lysogeny. The results are displayed for different *E. coli* sizes from 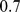, 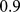 and 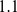. (B, C) The probability of lysogeny when the size of the host bacterium is 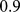 and 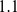 respectively. Black curve represents the behavior of a rational player. Blue dashed curve shows the behavior of bacteriophages from the intracellular view based on its regulatory network. Red circles show the behavior of bacteriophages based on the experimental data of [24].

**Figure 8:**
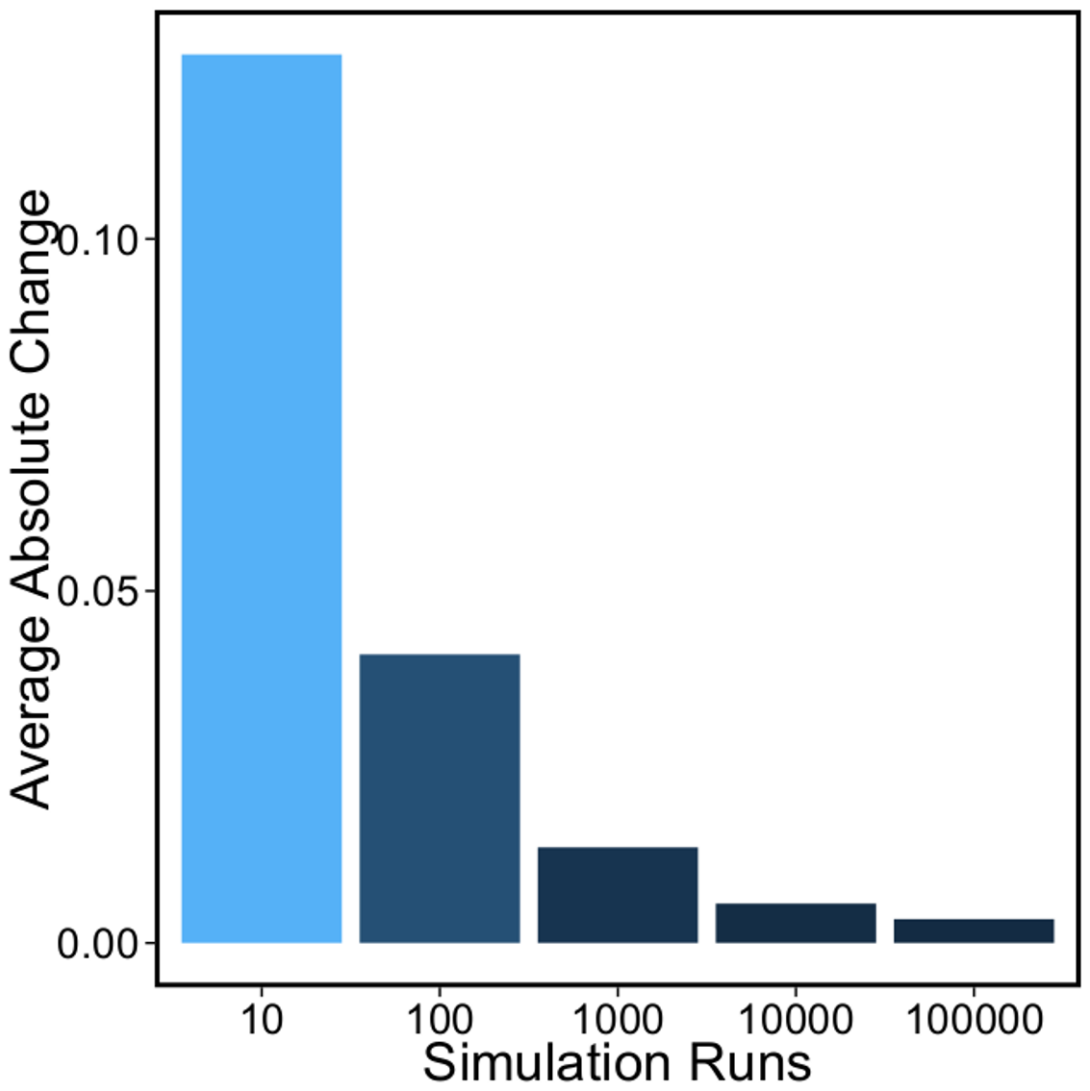
Number of simulation runs for robust results. We perform measurement of lysis/lysogeny probabilities with 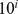 simulation runs where 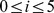. For each 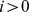, we computed the average absolute change in measured probability values from 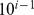 to 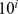 simulation runs (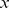-axis labels show 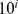). Our analysis shows 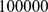 simulation runs are sufficient for producing robust results.

For every value of 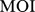 from 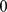 to 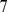 with 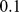 steps, we performed 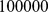 simulation iterations to compute the probability of lysis or lysogeny decision-making (Figure 7). In each iteration, we pick a random starting point in the landscape for the initial expression values of 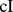 and 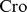, and track the trajectory of the cell towards different attractor states for a limited time, based on equation set 12. The commitment to either lysis or lysogeny is decided based on the final relative abundance of 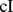 and 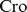 proteins (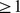 for lysogeny, 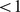 for lysis). For every value of MOI, the lysis or lysogeny probabilities are defined as the ratio of the trajectories that ended in each corresponding state (Figure 7). We selected 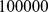 iterations since the probabilities do not significantly change between 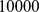 to 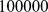 simulation iterations (see Figure 8). The commitment of the phage to either lysis or lysogeny is assigned based on the relative abundance of 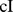 and 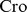 proteins (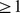 for lysogeny, 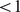 for lysis).

The trajectory path of the phage is divided into a fine mesh. The direction of the next step is computed based on the combination of the deterministic force field and the stochastic noise. For every value of 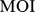, the ratio of the phages assigned to every outcome in the simulation is considered as the probability of every decision (Figure 7). The results of the simulation show a strong agreement with both experimental results reported in [24] and our expectation from a rational player reported in Figure 4.

To investigate the predictability of the model, the effect of the bacterium volume on the lysis/lysogeny decision is analyzed (Figure 7). In a population of bacteria with similar 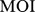, the computational model predicts that the smallest bacterium would have the highest probability of lysogeny. This is in agreement with in vitro experiments. It also shows that smallest volume bacterium, at the same 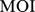, has the highest viral concentration. It has a positive effect on 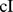 expression and increases the chance of lysogeny [24].

## Discussion

We have studied the decision-making problem of bacteriophages lambda after infecting the *E. coli* bacterium. In particular, we have proposed a model of rational decision making that considers phages as competitive and cooperative agents and where there exists a trade-off between lysogenic and lytic pathways. The lysogenic pathway maintains the host bacterium as a host for reproduction, while lytic pathway increases the number of bacteriophages in the environment and destroys the host bacterium [22]–[24], [28], [29], [37]. Thus, a rational decision maintains the host bacterium when there are enough bacteriophages in the environment and lysis otherwise. Making this decision requires external information from the environment which is represented by the average 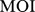. Such information is inferred based on internal signals such as size, 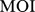, and concentration of the host bacterium [38]. Given these parameters, we have analyzed the behavior of a rational decision in various environmental situations (see Figure 3). Our results show that the behavior of a rational player matches that of bacteriophages as reported in the experimental results of [24], [30]–[32] (see Figure 4).

From an intracellular point of view, the probability distribution of the lysis/lysogeny decision, under different 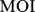 values, is defined by the structure of the gene regulatory network [30]–[35], [39]. In this context, mapping the structure of the gene regulatory network to the Waddington landscape gives comprehensive insight into the decision-making process. We trace how the environmental fluctuations reshape the landscape and change the fate of bacteriophages (see Figure 5 and Figure 6). We have argued that there exists an internal switch that makes decisions based on internal signals. We call it an internal biased coin that is encoded in the genome and inherited from its ancestors.

Moreover, internal signals affect the weights of the edges in the gene regulatory network which, in turn, determine the shape of the Waddington landscape, the position of stable points, and the behavior of bacteriophages. On the other hand, internal signals also influence the estimation of average 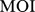 which defines the expected utility function, and consequently, the mixed strategy of a rational player. Our results demonstrate that internal signals determine both the weights of edges in the regulatory network and the estimation of average 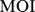 such that the probability distribution over attractors of the gene regulatory network is almost the same as the mixed strategy of a rational player (see Figure 7).

Overall, the gene regulatory network controls the decision making of individuals in a biological environment [17], [18]. Every cell makes decisions based on its intracellular genetic network and internal signals. The later determine the weights of the edges in gene regulatory network, and lead the organism to a stable point. A well-defined biochemical network, for decision making, is the one that can meet all of the cells requirements and changes the probability of their strategies based on different environmental situations. Mutations occur over time and change the structure of proteins, the shape of the regulatory network, the decision landscape, and the probability distribution of different decisions of an organism. Organisms with the highest adaptability will survive [19]. We have proposed a framework that shows how the gene regulatory network in the survivors leads the organism to behave almost the same as a rational player.

## Limitations of the Study, Open Questions, and Future Work

The proposed framework can be used to study various fundamental cellular decision-making problems, e.g. decision between two different pathways of ATP synthesis in yeast. We believe that in binary decision-making problems, there is a bistable network which determines the fate of the organism. Internal signals define both the weights of the edges of the bistable network from intracellular perspective, and the estimation of the environmental state considering them as competitor and cooperative organisms. Based on the proposed framework, the bistable network can be viewed as a biased coin that defines an almost optimal mixed strategy in the presence of cooperators and competitors. More precisely, our approach can be used for other cellular decision-making processes to quantitatively assess how the corresponding gene regulatory network affects the rational decision-making process in different isogenic cells.

We use sigmoid utility functions and the logit-response rule to model the rational player's decision-making process. Different utility functions and rules can be used for modeling decision-making processes. Studying the effect of different functions on decision-making models might be an appropriate direction for future studies. In addition, the gene regulatory dynamic equations presented in [22] are used as a basis for studying the decision-making process at the intracellular level, though other various mathematical models can also be used to represent the dynamic of a gene regulatory network in lysis/lysogeny decision making. Furthermore, the gene regulatory model we have used from [22] is limited to the triple key genes in lysis/lysogeny decision making. Investigating post-transcription modifications and translation noises that can cause deviations from quasi-steady state approximations of gene expression dynamics is a limitation of our study and can be studied further in the future.

## Conclusion

In this paper we present a framework to show how the decision making of isogenic cells corresponds to the underlying gene regulatory network from an intracellular perspective. We use a game theoretic approach to model the decision-making process, considering cells as cooperative and competitive agents. We also quantitatively model the underlying gene regulatory network and use the Waddington landscape for a comprehensive understanding of attractor states. We demonstrate that the attractor states of the corresponding Waddington landscape fit the strategies of a rational player in the corresponding game. We study a fundamental decision-making problem in *E. coli* where a decision between lysis/lysogeny is made. We show that a decision between lysis or lysogeny from an intracellular perspective, considering the gene regulatory networks, almost matches a rational decision which maximizes the chance of living. To conclude, we provide a framework that uses game theory on one end and gene regulatory networks on the other end to study various fundamental cellular decision-making problems. The proposed framework explains how cells make decisions similar to a rational player in an analogous game.

## Materials and Methods

### Mathematical model of a rational decision

To model the decision-making process of bacteriophages in *E. coli*, we first show how the environmental state can be estimated based on the internal information of phages such as 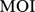 and size of the host bacterium. Then we present a method to determine the utility of each action and its corresponding probability based on the environmental state.

#### Determining environmental state

A phage inside *E. coli* senses the size and the multiplicity of infection by probing the concentration of proteins and the concentration of dimers such as 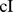 and 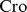 inside the host bacterium [33], [34]. Therefore, in our model, size and 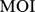 of the host bacterium are considered as internal signals.

Let the random variable 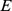 be the average 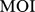 within a population of phages and bacteria, where the average 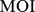 is the total number of phages infecting a bacterium over the total number of bacteria in the environment. Also let 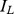 be the 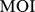 of a host bacterium with size 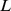, and 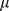 be the number of phages inside the host bacteria. A bacterium with size 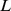 is a bacterium which its volume is 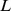 times the average volume of all bacteria in the environment A rational player infers about the average 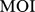 and defines posterior probability 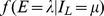 based on the Bayes rule:
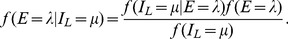
(1)


To compute the posterior probability, we estimate the value of 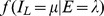, 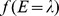, and 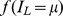. Consider an environment with average 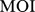 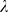 in which phages move and infect bacteria randomly. The infection process can be seen as a Poisson process with average rate 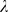 [36]. The Poisson distribution is a probability distribution that expresses the probability of a given number of events that occur in a fixed interval of time and/or space when the events occur with a known average rate [40]. On the other hand, the bacterium infection by phages is a random process, with an average number of infections 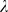, which is independent of previous infections. Hence, the probability distribution of 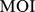 for a single bacterium of size 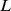, given the average 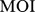 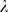, can be expressed by a Poisson distribution:
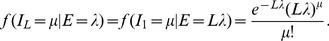
(2)


For prior probability 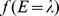 we assign a uniform distribution between 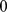 and 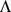, i.e., 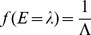 for 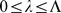. The above simple distribution enjoys the maximum entropy and equivalently the minimum knowledge among all priors. Uniform distribution is the most rational assumption when there is no prior information on the parameter [41], [42]. At the end, probability 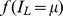 can be obtained by marginalizing out 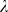 as follows:
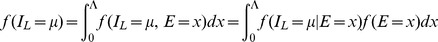


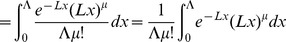


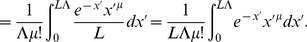
(3)


Now, probability 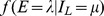 can be calculated based on equations 1, 2, and 3 as follows:
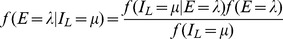


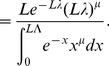
(4)


From the definition of gamma function, we have 
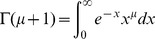
, where 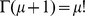 for a positive integer 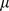 [43]. There is also a nice bound for the value of 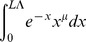 as follows:
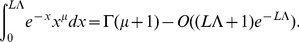
(5)


Equation 5 shows that 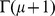 is a good estimation for 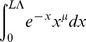. Putting all these facts together, we have:
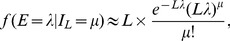
(6)

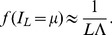
(7)


#### Utility Function

The lysis action increases the number of phages and kills the host bacterium. The lysogeny action only takes care of the host bacterium. Therefore, decision making in *E. coli* depends on the average 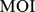. It means if the number of phages in the environment is high and there are not enough host bacteria, a rational player takes care of host bacterium and lysogenize. Otherwise, a rational player tends to lyse. The utility function is modeled with a threshold 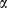. The utility of lysis overcomes the utility of lysogeny if and only if a rational player infers that the average 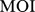 is less than 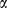.

To simplify the model, we look at the environmental state as a result of individual's strategies and define the utility functions based on the environmental state. We demonstrate the utility of individual 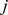 by function 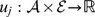. Note that individual's strategies affect the state of the environment in the future. Thus, in our model the utility function implicitly depends on other individual's strategies. Let 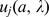 be the utility of action 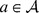 of individual 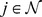 when the environmental state, average 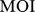, is 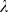. Let's assume the set of all possible actions is 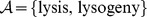. The utility of lysis should drop at threshold point 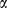 and the utility of lysogeny should rise at the same threshold point. This means the phages tend to keep the average 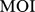 around 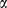 which is their desired average 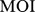. We focus on the sigmoid function and define the utility functions as follows:
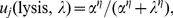
(8)

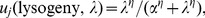
(9)


where 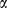 is a threshold point and phages are more likely to choose lysogenic pathway when their estimation about the average 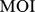 exceeds threshold 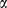 and 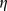 is a parameter that defines the slope of the sigmoid function.

Note that phages only detect internal signals, e.g., 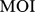 and size of the host bacterium and thus they need an estimation about the utility of each action based on the observed internal signals rather than the average 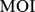. This is done by inferring about the average 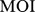 based on the internal signals. Therefore, the expected utility of action 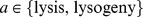 for a phage with host bacterium 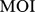 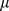 and host bacterium size 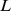, 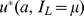, is defined by integrating over all possible environmental average 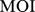
s as follows:
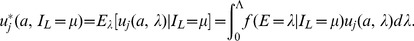
(10)


#### Strategies

There are several rules in the population games and evolutionary dynamics that determine the strategy of players based on their utility. We employ the noisy best-response rule, the logit-response rule, as a well-known rule in the discrete choice literature for environmental evolution that well matches our setting [44]–[47]. In the logit-response rule, every individual plays its best-response strategy with a probability close to 1. However, we allow a small possibility for making mistakes. Individuals might make mistakes in their inferences if their information about their surroundings are noisy or the agents are not entirely rational [44], [48]–[51]. In the logit-response rule the probability that individual 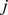 takes action 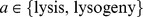, 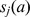, is proportional to 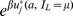 as follows:
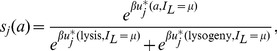
(11)where 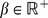 determines how noisy the system is. 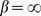 shows that the system is noise-free and every individual plays its best action and 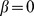 represents a full noisy environment in which every individual plays randomly. A value between these two extreme points is chosen for modeling the behavior of real-world decision makers [44], [48]–[51].

### Mathematical model of a gene regulatory network

The gene regulatory networks are mathematically represented in many different ways, including Boolean networks [52], Bayesian networks [53], ordinary differential equations (ODEs) [22], hybrid models [54], and even game theory [55]. Among all, we selected ODEs since they represent the dynamical states of small networks more precisely along the time.

The ordinary differential equations (ODEs) reported by [22] are used as a basis for the computational study of the lysis/lysogeny decision making at a molecular resolution.

A non-restrictive quasi-steady state approximation (QSSA) method is applied to reduce the number of equations based on the great difference in the rate of fast (binding and unbinding of proteins) versus slow reactions (transcription, translation and protein degradation):
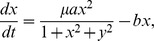


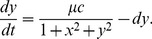
(12)


Here 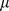 stands for multiplicity of infection (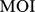), 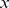 and 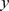 are the rescaled concentrations of 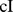 and 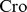 free monomers, 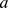 is transcriptional rate of 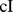 when the promoter is bound, 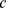 is the transcriptional rate of 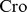 when the promoter is unbound, and 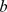 and 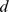 are degradation rates for 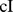 and 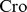 proteins respectively. More details about equations and parameters are provided in the supplementing information.

We model equation set 12 as a two-dimensional sample space, the force-field representation of which is provided in 5. Since this force-field representation is not a gradient vector fields, it is not possible to find an exact potential function for it. Therefore, we use a computationally feasible pseudo-potential function, only for a graphical representation of the landscape of the gene regulatory network. This potential function is monotonically increasing while it goes from an attractor center out to transient states (Figure 5). The logarithmic scale enables us to track the precise position of the attractor points. A pseudo-potential function is defined as follows:
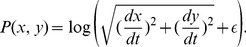
(13)


Here 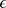 is a small positive value ensures that the logarithm function is computed for positive values. This equation assigns the minimum potential 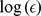 to all attractor states where 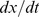 and 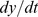 are zero. In theory, a similar potential function is assigned to unstable fixed points. However the vector-space representations of the force fields show such points are not present in our experiments (Figure 5, A–C).

## Additional Information

Programs for modeling both a rational decision and gene regulatory network have been written in C++ and 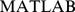.

## Supporting Information

Figure S1
**The root-mean-square error.** (A) This figure shows the root-mean-square error when 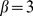 and 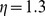 are fixed, and 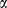 varies from 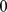 to 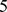. (B) This figure shows the root-mean-square error when 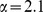 and 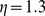 are fixed, and 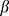 varies from 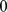 to 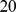. (C) This figure shows the root-mean-square error when 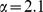 and 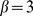 are fixed, and 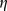 varies from 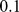 to 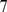.(TIFF)

Figure S2
**Lysogeny probability changes by different rate of** 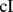 **dimerization.** Decreased dimerization rate of 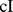 will decrease the lysogeny probability. A factor of 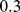 to the 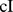 dimerization rate decreases the depth of lysogenic attractor (left). By a factor of 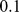, the lysogenic valley vanishes and only the lytic attractor remains (right).(TIFF)

Figure S3
**Alternations to the network structure will destruct the function of the genetic switch.** First, the negative effect of the 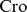 protein on the expression of its own gene is replaced by a positive effect. Second, the positive effect of 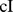 on its own gene is replaced by a negative effect.(TIFF)

Figure S4
**Promoter mutations can alter the decision landscape.** The mutations that increase the binding affinity of 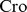 to the operator sites will deepen the lytic attractor (left), while the contrary mutations increase the chance of lysogeny (right).(TIFF)

Appendix S1 **Definition of parameters.** In this appendix we define parameters for modeling a rational decision and the Waddington model.
(PDF)

Appendix S2 **Verifying predictability of the Waddington model.** In this appendix we perform several analyses for confirming the predictability of the proposed Waddington model.
(PDF)

Video S1
**The effect of** 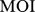 **on the Waddington landscape of phages in the lysis/lysogeny decision making.** This video describes how the positions of attractor points change in the Waddinton landscape, for the host bacterium 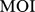 from 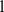 to 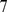.(MP4)
