# Supplementary figures and images for: Natural Biased Coin Encoded in the Genome Determines Cell Strategy
Source: PLoS One. 2014 Aug 4;9(8):e103569. doi: 10.1371/journal.pone.0103569 (PMC4121144; doi:10.1371/journal.pone.0103569)

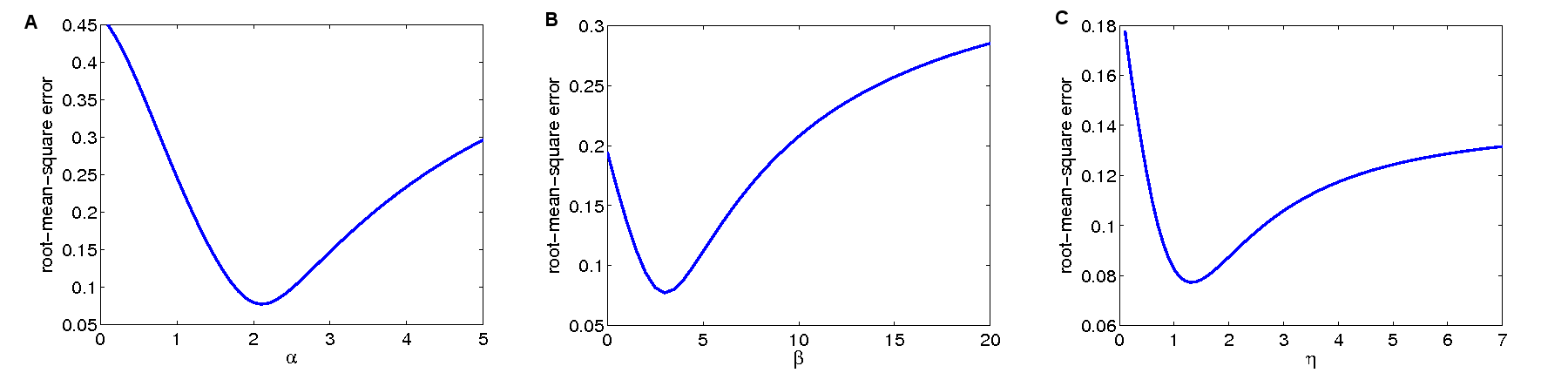

Supplement: Figure S1 — The root-mean-square error. (A) This figure shows the root-mean-square error when and are fixed, and varies from to . (B) This figure shows the root-mean-square error when and are fixed, and varies from to . (C) This figure shows the root-mean-square error when and are fixed, and varies from to . (TIFF) [file pone.0103569.s001.tif]

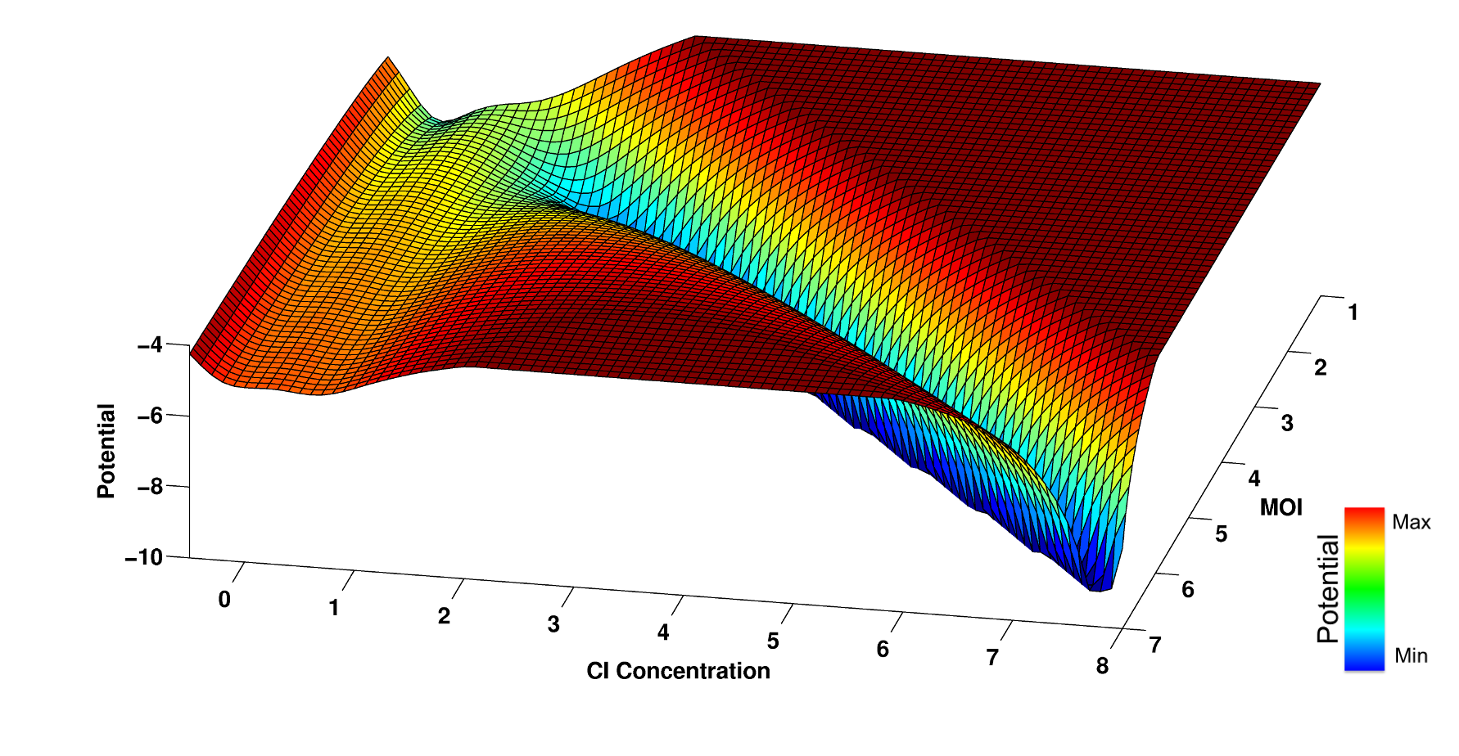

Supplement: Figure S2 — Lysogeny probability changes by different rate of dimerization. Decreased dimerization rate of will decrease the lysogeny probability. A factor of to the dimerization rate decreases the depth of lysogenic attractor (left). By a factor of , the lysogenic valley vanishes and only the lytic attractor remains (right). (TIFF) [file pone.0103569.s002.tif]

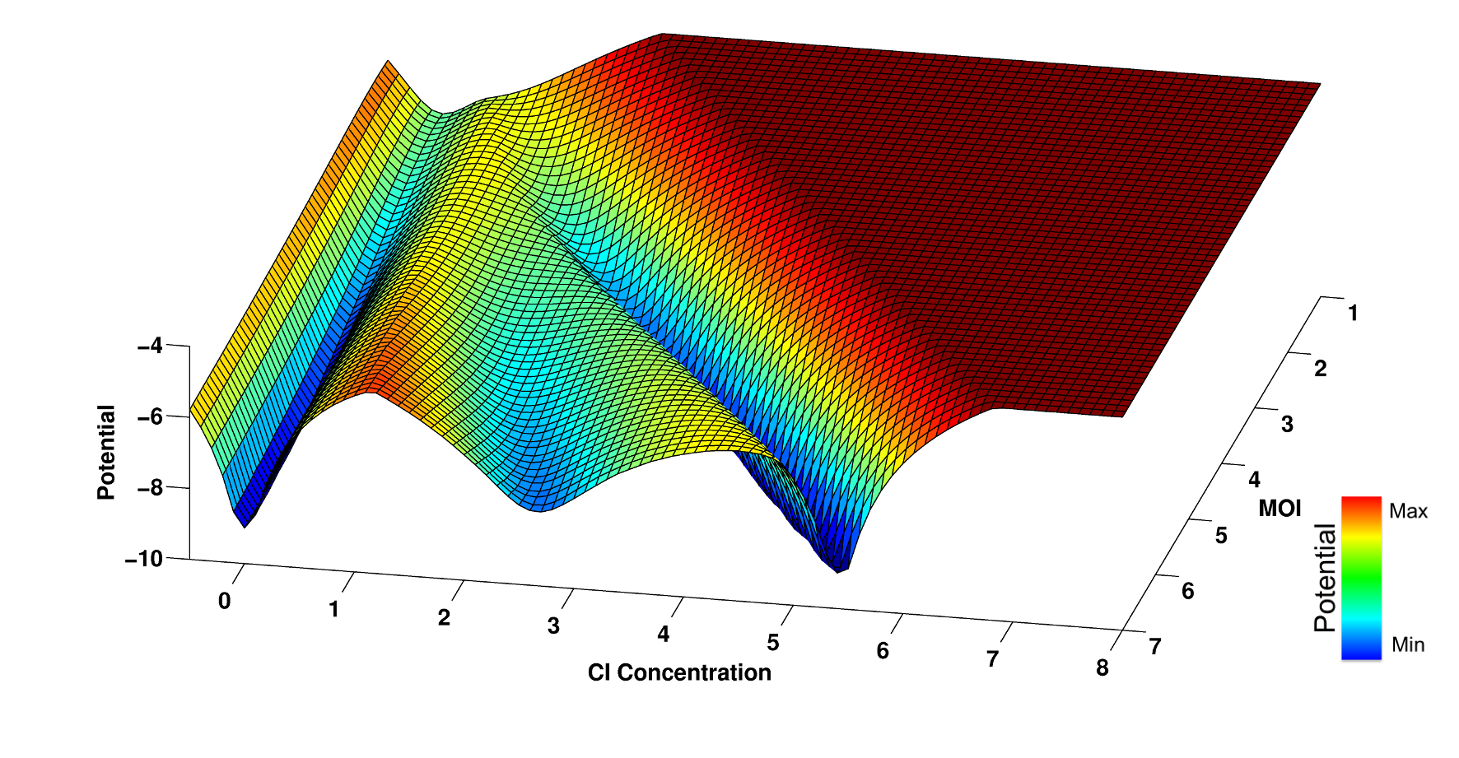

Supplement: Figure S3 — Alternations to the network structure will destruct the function of the genetic switch. First, the negative effect of the protein on the expression of its own gene is replaced by a positive effect. Second, the positive effect of on its own gene is replaced by a negative effect. (TIFF) [file pone.0103569.s003.tif]

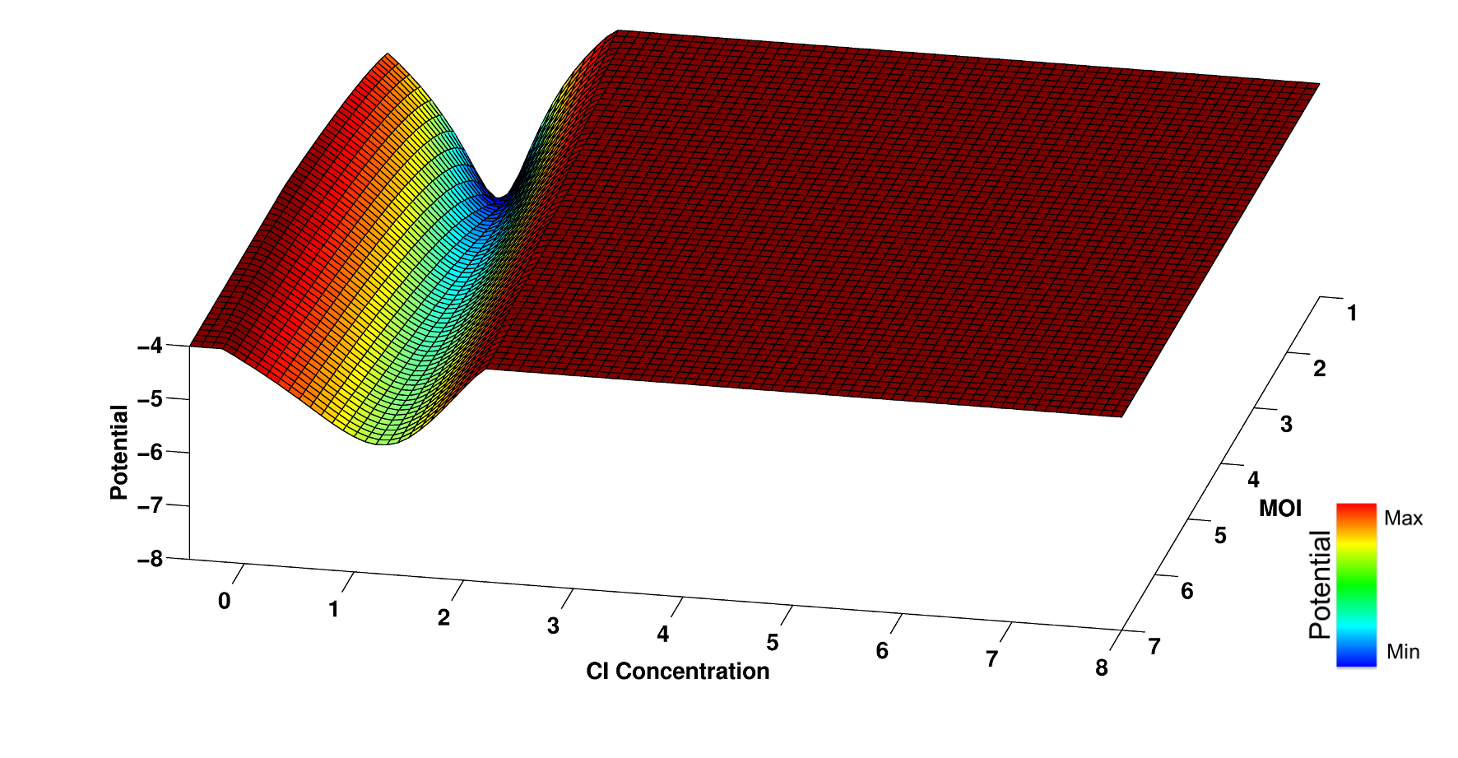

Supplement: Figure S4 — Promoter mutations can alter the decision landscape. The mutations that increase the binding affinity of to the operator sites will deepen the lytic attractor (left), while the contrary mutations increase the chance of lysogeny (right). (TIFF) [file pone.0103569.s004.tif]
