# Supplementary material for: Natural Biased Coin Encoded in the Genome Determines Cell Strategy
Source: PLoS One. 2014 Aug 4;9(8):e103569. doi: 10.1371/journal.pone.0103569 (PMC4121144; doi:10.1371/journal.pone.0103569)
Supplement: Appendix S1 — Definition of parameters. In this appendix we define parameters for modeling a rational decision and the Waddington model. (PDF) [file pone.0103569.s005.pdf]

Appendix S1 Definition of parameters

Parameters for modeling a rational decision

For modeling a rational decision, we should define parameters α, η, and β in equations 8, 9 and 11. Recall that α and η define the sigmoid functions. In particular, parameter α is a threshold point such that the phages are more likely to lysogenise when the estimated average MOI is greater that α, i.e., the ideal environmental MOI is α for the phages. Parameter η represents the slope of the sigmoid functions. This parameter shows the smoothness of the sigmoid functions. At last in the logit-response rule, parameter β is defined to capture the rationality of phages where extreme points 0 and ∞ represent a random decision and a fully rational decision respectively. Since there exist several limitations for making a fully rational decision in the real world, a value between extreme points is chosen for describing the behavior of decision makers.

We employ the root-mean-square error (RMSE) for choosing the parameters which better match the experimental data of [2]. We have written a program for finding the parameters which minimizes RMSE and have found optimal values $\alpha = 2.1$, $\eta = 1.3$, and $\beta = 3$ with RMSE equals 0.077. Figure S1 shows the RMSE for different values of α, β, and η. Note that the results of Figure 4 and Figure 7 are based on these optimal values. These values do not affect the general shape of our lysogeny probabilities and results remain unchanged with another rational values of α, η, and β.

Parameters in the Waddington model

The equation set 12 comes with two equations that reflect dynamics of concentrations of free monomers of two proteins cI and Cro. Each equation consists of two terms: one for synthesis and the other for degradation of each protein. Each phage genome in a single bacterium is a basement for synthesis of the two proteins, so the synthesis terms of both equations has a factor μ as the multiplicity of infection (MOI). The protein cI is most expressed when its promoter is bound by the dimer cI_2, so there is a term ax^2 in synthesis term of the first equation, that reflects concentration of cI_2 dimers (x^2) multiplied to a constant rate of synthesis of cI when its promoter is bound (a). Also the main expression state of Cro is when its promoter is unbound, which is reflected by a constant synthesis rate c in the second equation.

The $x^2 + y^2 + 1$ divisor of cI synthesis equation adds to the model the negative effect of increased Cro_2 concentration (y^2) on expression of cI, while the effect of increased cI_2 concentration remains positive (because of x^2 in dividend). The same divisor in Cro synthesis equation imports a negative feedback of both cI_2 and Cro_2 concentrations to the expression of Cro. The unit in divisor prevents division by zero. Also the whole synthesis terms are sigmoid shaped functions that match the natural behavior in expression of many genes as a response to some other variable - like concentration of a transcription factor.

Based on the kinetic values reported in [1], the following approximation values are used in the equations: $a = 0.08\ min^{-1}$, $b = 0.1\ min^{-1}$, $c = 0.04\ min^{-1}$, $d = 0.05\ min^{-1}$

References

1. Weitz JS, Mileyko Y, Joh RI, Voit EO (2008) Collective decision making in bacterial viruses. Biophys J 95: 2673-2680.

2. Zeng L, Skinner SO, Zong C, Sippy J, Feiss M, et al. (2010) Decision making at a subcellular level determines the outcome of bacteriophage infection. Cell 141: 682-691.
