# Supplementary material for: Natural Biased Coin Encoded in the Genome Determines Cell Strategy
Source: PLoS One. 2014 Aug 4;9(8):e103569. doi: 10.1371/journal.pone.0103569 (PMC4121144; doi:10.1371/journal.pone.0103569)
Supplement: Appendix S2 — Verifying predictability of the Waddington model. In this appendix we perform several analyses for confirming the predictability of the proposed Waddington model. (PDF) [file pone.0103569.s006.pdf]

Appendix S2 Verifying predictability of the Waddington model

We have already shown that the computational model of lysis/lysogeny based on Waddington's approach is predictive for different sizes of the bacterium. In order to confirm the predictability of this model, we performed several other analyses mentioned below.

Lytic induction in lysogenic cells during severe survival conditions is another event to analyze the integrity of the Waddington decision model. The genome of the phage is replicated in lysogeny phase via the bacterial cell divisions. Undesirable signals about cell survival like damaged DNA activate the RecA protein, a key enzyme of homologous recombination, to initiate the SOS defense response system and repair the DNA. This uncertain situation of the bacterium survival is risky for the phage to be silent. Therefore, the cleavage of cI dimer by RecA occurs and activation of Cro promotes the lytic phase [2,3]. Reducing cI dimerization rate in the computational model made the lysogeny attractor state to disappear and increased the probability of lysis (Figure S2) that is another support for the model. It also shows how the strategy of the phage can rapidly adopt the alternations of the environment through a fast decision.

How the lysis/lysogeny probability distribution is inherited From a systems biology perspective, the outcome of a genetic switch depends on two factors: The network structure of the regulatory interactions between the genes, and the strength of the interactions. We first checked the particular network structure between cI and Cro, consisting of mutual repression of the two genes, self-repression of Cro and self-activation of cI. Replacing the negative loop of the Cro protein on its own expression with a positive loop (self-activation) would result in appearance of a third stable steady state, shown as the middle valley in Figure S2, in which both cI and Cro were expressed and so could not lead the phage a firm selection of either lysis or lysogeny. On the other hand the negative feedback of cI on its own expression would reform the landscape monostable with a unique lysis attractor (see Figure S3) that will void the switching function. Replacing the mutual repression between the two genes with positive interactions would also have similar consequences. So according to a Waddington perspective, the structure of the regulatory network between the two genes has well established to afford the desired function.

We next altered the strength of the interactions, as the second important factor in the outcome of the genetic switch. The strength of the regulation induced by a DNA binding protein on the expression of a target gene depends on how likely the protein is bound to the regulatory region of that gene. Mutations in the three operator sites OR_1, OR_2 and OR_3 are shown to alter the affinities and patterns of binding cI and Cro to the promoter region [1,4]. Our results show alternations in binding affinities that favor Cro binding to the promoter site would deepen the lytic attractor and decrease the lysogeny probability. In contrast, increasing the binding affinity of cI to the promoter site and decreasing binding affinity of Cro deepened the lysogenic attractor (see Figure S4).

References

1. Cao Y, Lu H, Liang J (2010) Probability landscape of heritable and robust epigenetic state of lysogeny in phage lambda. Proc Natl Acad Sci USA 107: 18445–18450.

2. Sauer RT, Ross MJ, Ptashne M (1982) Cleavage of the lambda and P22 repressors by recA protein. J Biol Chem 257: 4458-4462.

3. Galkin VE, Yu X, Bielnicki J, Ndjonka D, Bell CE, et al. (2009) Cleavage of bacteriophage λ cI repressor involves the RecA C-terminal domain. J Mol Biol 385: 779-787.

4. Little JW, Shepley DP, Wert DW (1999) Robustness of a gene regulatory circuit. EMBO J 18: 4299-4307.
